# Integrative analysis of genomic and epigenomic regulation of the transcriptome in liver cancer

**DOI:** 10.1038/s41467-017-00991-w

**Published:** 2017-10-10

**Authors:** Hyun Goo Woo, Ji-Hye Choi, Sarah Yoon, Byul A. Jee, Eun Ju Cho, Jeong-Hoon Lee, Su Jong Yu, Jung-Hwan Yoon, Nam-Joon Yi, Kwang-Woong Lee, Kyung-Suk Suh, Yoon Jun Kim

**Affiliations:** 1 Department of Physiology, Ajou University School of Medicine, 164 Worldcup-ro, Yeongtong-gu, Suwon, 16499 Republic of Korea; 2 Department of Biomedical Sciences, Graduate School, Ajou University, 164 Worldcup-ro, Yeongtong-gu, Suwon, 16499 Republic of Korea; 3 Department of Internal Medicine, Liver Research Institute, Seoul National University College of Medicine, 28 Yongon-dong, Chongno-gu, Seoul, 110-744 Republic of Korea; 4 Department of Surgery, Seoul National University College of Medicine, 28 Yongon-dong, Chongno-gu, Seoul, 110-744 Republic of Korea

## Abstract

Hepatocellular carcinoma harbors numerous genomic and epigenomic aberrations of DNA copy numbers and DNA methylation. Transcriptomic deregulation by these aberrations plays key driver roles in heterogeneous progression of cancers. Here, we profile DNA copy numbers, DNA methylation, and messenger RNA expression levels from 64 cases of hepatocellular carcinoma specimens. We find that the frequencies of the aberrancies of the DNA copy-number-correlated (CNVcor) expression genes and the methylation-correlated expression (METcor) genes are co-regulated significantly. Multi-omics integration of the CNVcor and METcor genes reveal three prognostic subtypes of hepatocellular carcinoma, which can be validated by an independent data. The most aggressive subtype expressing stemness genes has frequent *BAP1* mutations, implying its pivotal role in the aggressive tumor progression. In conclusion, our integrative analysis of genomic and epigenomic regulation provides new insights on the multi-layered pathobiology of hepatocellular carcinoma, which might be helpful in developing precision management for hepatocellular carcinoma patients.

## Abstract (web summary)

Hepatocellular carcinoma is known to harbour numerous genomic and epigenomic aberrations, driving transcriptomic deregulation. Here, the authors integrate genomic, epigenomic, and expression data to reveal three prognostic subtypes, providing insight to the pathobiology of hepatocellular carcinoma.

## Introduction

Recent large-scale and multi-omics profiling of cancers has provided a systematic picture of genomic and epigenomic deregulation in these diseases. Genomic alterations due to DNA copy-number aberration or mutations occur frequently during tumorigenesis, stimulating cancer progression. Epigenetic regulation of the cancer genome by DNA methylation also plays pivotal roles in heterogeneous cancer behaviors. In particular, in hepatocellular carcinoma (HCC), genomic profiling studies have demonstrated the enormous heterogeneity of genomic and epigenomic deregulation^1^. In this cancer, aberrations of DNA copy number play key regulatory roles in HCC progression^2–4^, and transcriptional deregulation resulting from such aberrations is a potential driver event in HCC progression^5, 6^. In addition, DNA methylation profiling studies have revealed the biological and clinical significance of epigenetic regulation in HCC progression^7–11^. Several key cancer-related genes such as *IGF2* ^12^ and *UHRF1* ^13^ exert their regulatory functions by modulating DNA methylation.

However, despite the genome-wide impact of aberrations of DNA copy numbers and DNA methylation on cancers, it remains unclear whether DNA copy-number aberration is systematically related to epigenetic DNA methylations, and, if so, whether this connection plays any role in cancer progression. In this study, we profiled DNA copy numbers, DNA methylation, and messenger RNA (mRNA) expression levels in a cohort of HCC patients. To identify genes whose expression levels are regulated by genomic and/or epigenomic deregulation, we defined DNA copy-number-correlated (CNVcor) and DNA methylation-correlated (METcor) genes, based on their corresponding gene expression levels across samples, respectively. CNVcor genes represent the transcriptional deregulation dependent on DNA copy number, whereas METcor genes represent transcriptional deregulation dependent on DNA methylation. Expression of CNVcor genes was significantly correlated with expression of METcor genes, suggesting concomitant regulation of cancer transcriptomes by alterations in genomic DNA copy number in addition to epigenetic DNA methylation. Moreover, by performing multi-omics integration of CNVcor and METcor genes, we could identify distinct molecular subtypes that were significantly associated with prognostic outcomes of HCC. Further systematic analysis could identify new mutations that could be used as precision targets or biomarkers for subtype distinction.

## Results

### Transcriptome deregulation by DNA copy number or methylation

**Fig. 1:**
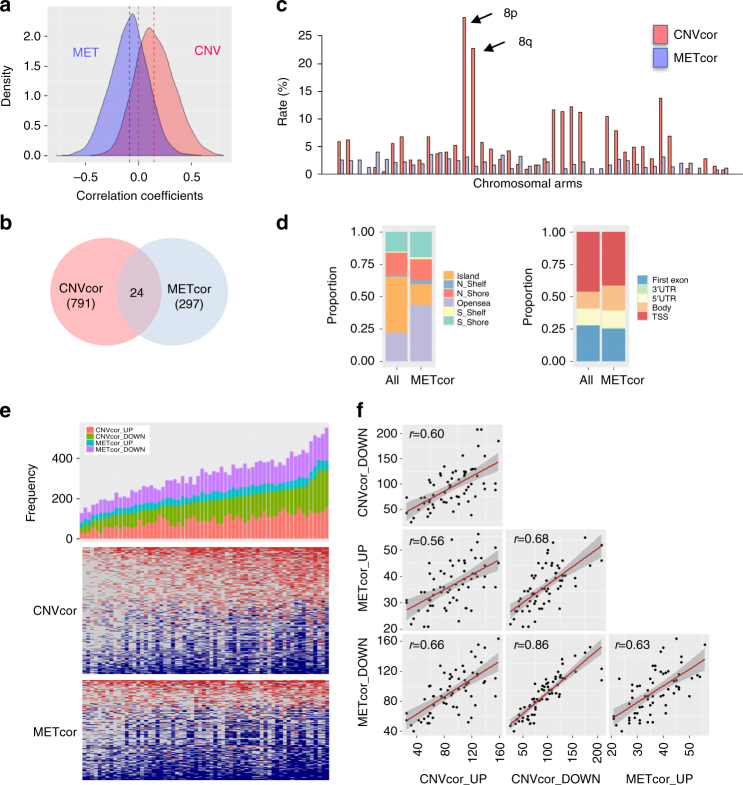
Identification of DNA copy-number-correlated (CNVcor) and DNA methylation-correlated (METcor) genes in HCC. **a** Distribution of the correlation coefficients between the mRNA expression levels and DNA copy numbers or DNA methylation across the samples are shown, respectively. **b** A Venn diagram shows the counts of CNVcor and METcor genes. The counts of overlapped genes between CNVcor and METcor are indicated. **c** Proportional frequencies of the CNVcor or METcor genes against total gene counts in each chromosome arm are shown, respectively. **d** Genomic positions of DNA methylation probes are categorized based on the positional relations with CpG islands (right) and genes (left), respectively. The proportional frequencies in each category of DNA methylation for the whole genes and METcor genes are compared, respectively. **e** A barplot shows the frequencies of up- or down-expressed CNVcor genes or METcor genes in each sample (top). Each of CNVcor and METcor genes is categorized as upregulated (CNVcor_UP and METcor_UP) or downregulated (CNVcor_DOWN and METcor_DOWN) genes, respectively. Heatmaps show the up- (red) and down-expressed (blue) CNVcor (middle) and METcor (bottom) genes, respectively. **f** Dot plots show the pairwise correlations among the frequencies of CNVcor_UP, CNVcor_DOWN, METcor_UP, and METcor_DOWN genes, respectively

Genomic and epigenomic profiles of DNA copy-number variation (CNV), DNA methylation (MET), and gene expression (EXP) were obtained from 64 HCC patients. Raw data profiles were preprocessed as described in “Methods.” To assess the global effects of genomic and/or epigenomic aberrations, we calculated the correlation coefficients between DNA copy number or DNA methylation profiles with the corresponding mRNA expression profiles. The correlation coefficient *r* was normalized to stabilize variance by applying Fisher’s *Z*-transformation: $z = \frac{1}{2}{\rm{ln}}\left( {\frac{{1 + r}}{{1 - r}}} \right)$. Consistent with previous studies, the overall distribution of correlation coefficients between DNA copy numbers and the corresponding gene expression profiles exhibited a significant skew to the right (skewness = 0.224, *P* < 2.2 × 10^−16^, D’Agostino test). By contrast, the correlation coefficients between DNA methylation profiles and corresponding gene expression profiles were skewed to the left (skewness = −0.153, *P* *=* 7.9 × 10^−11^) (Fig. 1a). This result reflects the overall impacts of positive and negative transcriptional deregulation due to aberrant DNA copy numbers and DNA methylation, respectively.

We next identified the positively correlated gene signatures for DNA copy number (CNVcor, *n* = 813) and the negatively correlated gene signatures for DNA methylation (METcor, *n* = 321), which were determined based on the Fisher’s *Z*-transformed correlation coefficients with 95% confidence interval ( ≥ 1.96 or ≤ −1.96, *P* < 0.05). (Supplementary Data 1). CNVcor genes exhibited DNA copy-number-dependent transcriptional deregulation, whereas METcor genes exhibited DNA methylation-dependent transcriptional deregulation. The CNVcor and METcor were not likely to overlap each other showing only 24 genes were overlapped, which may imply exclusive regulation of CNVcor and METcor genes in transcriptional deregulation (Fig. 1b). The CNVcor genes exhibited a regional genomic preference for DNA copy-number aberration, particularly on the *p* and *q* arms of chromosome 8 (Fig. 1c). Consistent with previous studies, the abundance of the CNVcor genes on chromosome 8 indicated regional sensitivity of gene expression to DNA dosage^5, 14^. By contrast, the METcor genes were not distributed in preferred chromosome regions. Moreover, in comparison with all probes, METcor genes frequently resided in the inter-genic open sea regions rather than CpG islands (Fig. 1d, left). In relation to genic position, DNA methylation was more frequent in gene body regions than near transcription start site (TSS) or first-exon regions (Fig. 1d, right), indicating that methylation in open sea and/or gene body regions might function in transcriptional deregulation. In addition, gene ontology analysis revealed differential enrichment of gene functions between CNVcor and METcor genes. CNVcor genes were enriched in functions related to protein transport (enrichment score, ES = 4.1), metabolism (ES = 3.88), and the cell cycle (ES = 2.29), whereas METcor genes were enriched in functions related to inflammation (ES = 3.41) and metabolism (ES = 3.12) (Supplementary Fig. 1). Although further studies are required, our results imply that CNVcor and METcor genes play distinct functional roles in transcriptional deregulation of HCC.

Because the CNVcor and METcor genes were thought as the aberrations with concomitant transcriptional deregulation, we further interrogated the association between the expression of the CNVcor and METcor genes by calculating the frequencies of transcriptional deregulation of both types of genes in each patient. This analysis revealed that patients with frequent deregulation of the CNVcor genes were more likely to exhibit frequent deregulation of the METcor genes (Fig. 1e). In addition, we designated up and downregulated CNVcor (CNVcor_UP and CNVcor_DOWN) and METcor (METcor_UP and METcor_DOWN) genes whose expression levels differed by more than twofold between tumors and surrounding non-tumoral tissues. Pairwise comparison of the frequencies of up- and down-regulated CNVcor or METcor genes revealed significant correlations between the two sets (*P* < 0.001, Pearson’s correlation test, Fig. 1f). Thus, we suggest that the frequencies of DNA copy-number- and DNA methylation-dependent transcriptional deregulation were highly correlated with each other.

### Molecular subtypes based on CNVcor and METcor genes

**Fig. 2:**
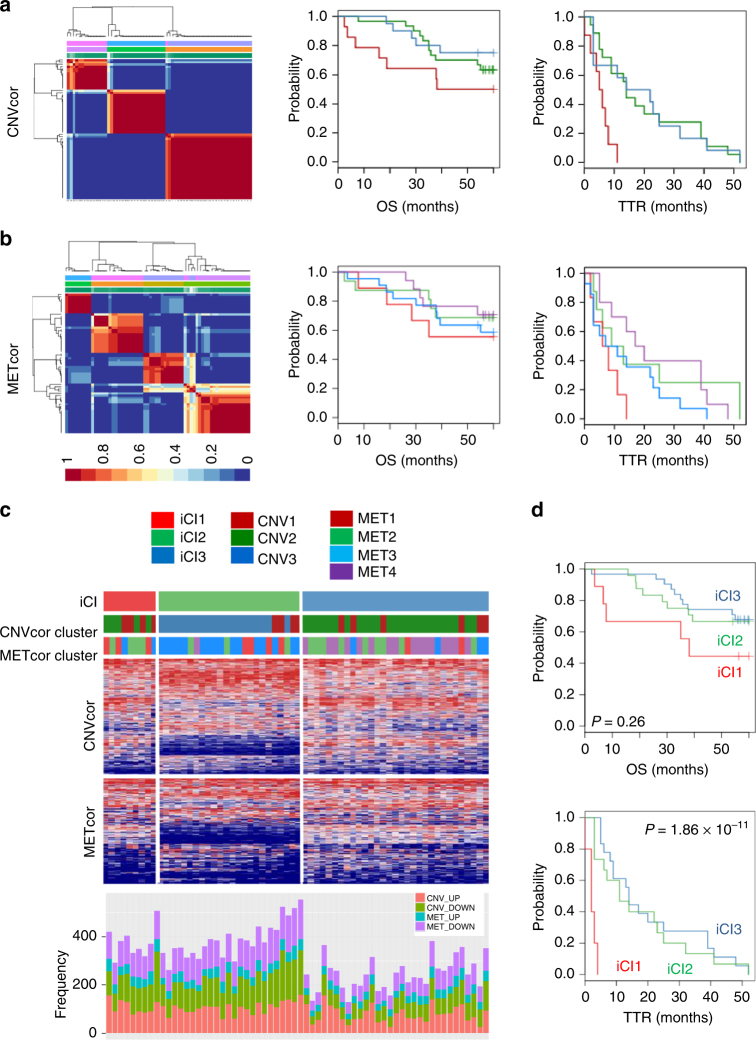
Identification of molecular subtypes of HCC using CNVcor and METcor genes. **a**, **b** Plots show the non-negative factorization (NMF) cluster results for the CNVcor in CNV data (**a**) and for the METcor in MET data (**b**), respectively. Kaplan–Meier plot analyses for subtypes identified by NMF clustering of the CNVcor and METcor genes are shown for overall survival (OS) and time to tumor recurrence (TTR), respectively. **c** Heatmaps show the expression patterns of subtypes identified by iCluster analysis. The subtypes identified by the CNVcor or METcor genes using NMF cluster methods are indicated with colored bars (top). The frequencies of aberrant expression of CNVcor_UP, CNVcor_DOWN, METcor_UP, and METcor_DOWN genes in each subtype are shown (bottom). **d** Kaplan–Meier plot analyses for subtypes identified by iCluster (iCl1, iCl2, and iCl3) are shown for overall survival (OS) and time to tumor recurrence (TTR), respectively

Next, we investigated whether the expression profiles of CNVcor and METcor genes could predict prognostic subgroups. For each gene set profile, we applied a non-negative matrix factorization (NMF) cluster analysis with cluster number *k* from 2 to 5, and then determined *k* for each profile: CNV, *k* = 3; MET, *k* = 4 (Fig. 2 and Supplementary Fig. 2). Interestingly, the subtypes identified by CNVcor overlapped significantly with the subtypes identified by METcor (*P* = 1.36 × 10^−5^, *χ* ^2^-test), consistent with the associated regulation of CNVcor and METcor genes in HCC. In addition, Kaplan–Meier plot analyses demonstrated that the subtypes identified by CNVcor or METcor genes could predict patients’ clinical outcomes of time to recurrence (TTR), respectively (CNVcor: TTR, *P* = 0.004; METcor: TTR, *P* = 0.07). Kaplan–Meier plot of overall survival (OS) also revealed distinct prognostic outcomes among the groups, although the difference was not statistically significant (CNVcor: OS, *P* = 0.22; METcor: OS, *P* = 0.7), possibly due to the smaller sample size in each subtype.

We next sought to identify molecular subtypes that reflected the multi-layered expression patterns of the CNVcor and METcor genes. To integrate the genomic data regarding DNA copy numbers, DNA methylation, and RNA expression, we employed an integrated cluster method, iCluster^15^. Clustering analysis was performed with cluster numbers *k* *=* 3 and 4, which were determined as the number of clusters from the NMF analyses on the individual data sets, respectively. This analysis revealed three distinct subtypes with *k* = 3, iCl1, iCl2, and iCl3, which were consistent with the classification based on the NMF analysis for individual CNV and MET data, respectively (*P* = 3.65 × 10^−11^ and 1.09 × 10^−5^; *χ* ^2^-test). Interestingly, the frequencies of aberrant CNVcor and METcor genes were much higher in subgroups iCl1 and iCl2 than in iCl3 (Fig. 2c, bottom), indicating that the subtypes were distinct from one other concerning the correlated regulation of transcriptomes by genomic and epigenomic aberrations.

Notably, Kaplan–Meier plot analysis revealed that, among the three subgroups, iCl1 had the worst prognostic outcomes of TTR and OS (TTR, *P* = 1.86 × 10^−11^; OS, *P* = 0.26, Fig. 2d). We also compared clinico-pathological features among the iCl1, iCl2, and iCl3 subgroups, and found that iCl1 tumors had more frequent vascular invasion than iCl2 or iCl3 tumors (*P* = 0.002, Table 1). Other clinical features did not differ among the subgroups. These results are consistent with the distinct aggressiveness properties of the molecular subtypes. Based on these findings, we suggest that integrated analysis of CNVcor and METcor genes can identify molecular subtypes, each of which features a different combination of genomic and epigenomic features related to transcriptional deregulation, associated with distinct prognostic outcomes.

**Table 1:** Clinico-pathological features of the molecular subtypes of SNU data

	iCl1 (*n* = 9)	iCl2 (*n* = 24)	iCl3 (*n* = 31)	*P* value (*χ* ^2^-test)
*Sex*				
Male	8	19	29	0.276
Female	1	5	2	
*Age*				
<60 (years)	5	12	16	0.960
≥60 (years)	4	12	15	
*Stage*				
T1/T2	5	21	27	0.065
T3/T4	4	3	4	
*Nodal metastasis*				
No	7	24	30	0.060
Yes	1	0	0	
*Tumor size*				
**<**5 cm	6	10	12	0.319
≥5 cm	3	14	19	
*Vascular invasion*				
No	1	15	19	^a^0.002
Yes	7	16	5	
*Gross type*				
Simple nodular	3	16	14	0.110
Multi nodular/infiltrative	6	7	16	
*AFP*				
<400 (ng/ml)	5	18	25	0.286
≥400 (ng/ml)	4	5	6	
*Grade*				
I, II	2	11	19	0.278
III, IV	4	5	10	

^a^Statistical significance *P* < 0.05

### Validation of the molecular subtypes in an TCGA data set

**Fig. 3:**
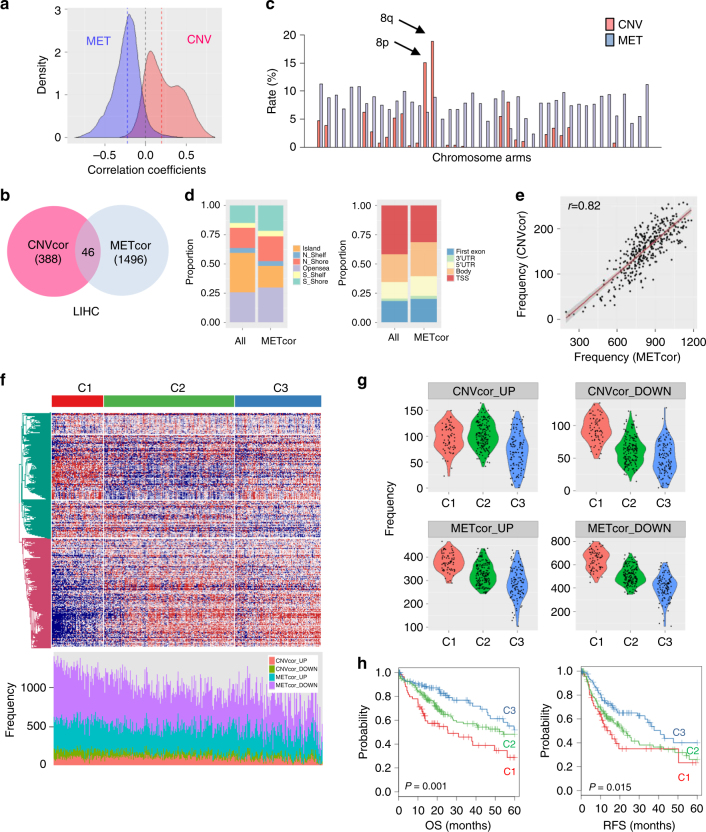
Validation of molecular subtypes using an independent TCGA data. **a** Overall distributions of correlation coefficients of between the mRNA expression levels and DNA copy numbers or DNA methylation are shown, respectively. **b** A Venn diagram shows the counts of the DNA coy number-correlated genes (CNVcor) and the DNA methylation-correlated genes (METcor) genes. The counts of the overlapped genes between CNVcor and METcor are indicated. **c** Proportion of the CNVcor or METcor genes against total count of genes in each chromosome arm are shown. **d** Composition of DNA methylation probes in the whole genes and METcor genes are compared. Genomic positions of DNA methylation probes are categorized based on the relations with CpG island regions (right) and gene regions (left), respectively. **e** A plot shows the correlation between the frequencies of CNVcor and METcor genes in each sample. **f** A heatmap shows the expression patterns of the differentially expressed genes among the subtypes identified by iClusterPlus analysis (top). The frequencies of CNVcor_UP, CNVcor_DOWN, METcor_UP, and METcor_DOWN genes are shown (bottom). **g** Violin plots indicate the frequencies of CNVcor_UP, CNVcor_DOWN, METcor_UP, and METcor_DOWN genes are shown in each subtype of C1, C2, and C3, respectively. **h** Kaplan–Meier plot analyses of the HCC subtypes for overall survival (OS) and recurrence-free survival (RFS) are shown, respectively

To validate the robustness and consistency of our molecular classification using the CNVcor and METcor genes, we applied the same method to an independent HCC data set from TCGA (LIHC, Liver Hepatocellular Carcinoma). Consistent with the aforementioned analysis, in the LIHC data, we observed that the overall distribution of correlation coefficients between the CNV and transcriptome was shifted to the right (skewness = 0.367, *P* < 2.2 × 10^−16^, D’Agostino test), whereas the correlation coefficients for DNA methylation were shifted to the left (skewness = −0.237, *P* < 2.2 × 10^−16^) (Fig. 3a). CNVcor (*n* = 388) and METcor (*n* = 1496) genes were identified from the LIHC data using the same analysis pipeline (Fig. 3b). Consistent with the Seoul National University (SNU) data, CNVcor genes were enriched on chromosome 8, whereas the METcor genes did not exhibit a chromosomal preference (Fig. 3c). METcor genes were more frequent in open sea and gene body regions than in CpG islands and TSS regions (Fig. 3d). In addition, the correlation between the frequencies of aberrant CNVcor and METcor genes was validated in the LIHC data (*r* = 0.82, *P* = 1.69 × 10^−88^, Fig. 3e).

In addition, we determined the molecular subtypes of LIHC data using the same method that we applied to the SNU data. This analysis also revealed three subtypes (C1, C2, and C3), which had clear differences in their frequencies of CNV and methylation aberrations (Fig. 3f). Group C1 had the highest rate of aberrations in DNA copy number and DNA methylation, whereas group C3 had the lowest rate (Fig. 3f, g). As expected, the three groups had distinct prognostic outcomes, with the worst OS (*P* = 0.001) and recurrence-free survival (RFS, *P* = 0.015) in group C1, and the most favorable clinical outcomes in the group C3 (Fig. 3h). These results confirmed that our strategy for classifying tumors based on CNVcor and METcor can identify prognostic subgroups with distinct genomic and epigenomic regulation that reflect the clinical outcomes of HCC patients. Thus, the profile of CNVcor and METcor genes can predict prognostic molecular subtypes independent of patient cohort and data platforms.

### Coordinated aberrations of DNA copy numbers and methylation

**Fig. 4:**
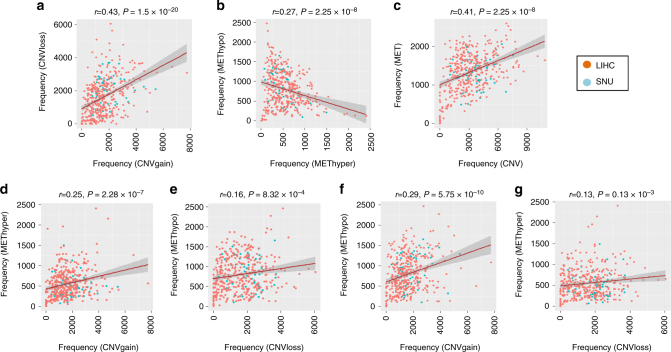
Coordinated aberrations of DNA copy number and DNA methylation in liver cancer. **a**, **b** The SNU and LIHC data sets of DNA copy numbers or DNA methylation are integrated by applying “combat” method as described in “Methods.” Aberrations of DNA copy numbers or DNA methylation were determined with cutoff fold difference >0.2 compared to those of the average values of the non-tumoral tissues, respectively. Directional alterations of DNA copy-number gain (CNVgain) and loss (CNVloss) and DNA hypermethylation (METhyper) and hypomethylation (METhypo) in each sample are plotted, respectively. **c** Overall frequencies of CNV aberration including CNVgain and CNVloss and DNA methylation including METhyper and METhypo are plotted. **d**–**g** Plots show the pairwise frequencies of CNVgain, CNVloss, METhyper, and METhypo genes in individual samples

We also compared the frequencies of the aberrant DNA copy number and DNA methylation in the entire genome in the integrated SNU and LIHC data set (*n* = 428) after correcting for batch effects as described in “Methods.” The DNA copy-number gain (CNVgain) and loss (CNVloss) and the DNA hypermethylation (METhyper) and hypomethylation (METhypo) were determined with the cutoff of the fold difference 0.2 compared to the average value for each probe of non-tumoral tissues. The frequencies of CNVgain were significantly correlated with the frequencies of CNVloss (*r* = 0.43, *P* = 1.5 × 10^−20^, Fig. 4a). By contrast, the frequencies of DNA hypermethylation were negatively correlated with the frequencies of DNA hypomethylation (*r* = −0.27, *P* = 2.25 × 10^−8^, Fig. 4b). In addition, as with the frequencies of CNVcor and METcor aberrations, the frequencies of the aberrant DNA copy number, including CNVgain and CNVloss, were significantly correlated with the frequencies of aberrant DNA methylation, including METhyper and METhypo (*r* = 0.41, *P* = 2.25 × 10^−18^, Fig. 4c). Directional aberrations of CNVgain and CNVloss, and METhyper and METhypo, were also closely correlated, indicating that the correlations were independent of directional changes (Fig. 4d–g). Taken together, we suggest that the HCC patients with frequent aberration of DNA copy number are more likely to have frequent aberration of DNA methylation. These correlated frequencies of aberrant CNVcor and METcor genes may imply the close relationships between the aberrations of DNA copy number and DNA methylation, and which were not dependent to patient cohorts or data platforms.

### Identification of genomic key features in the HCC subtypes

**Fig. 5:**
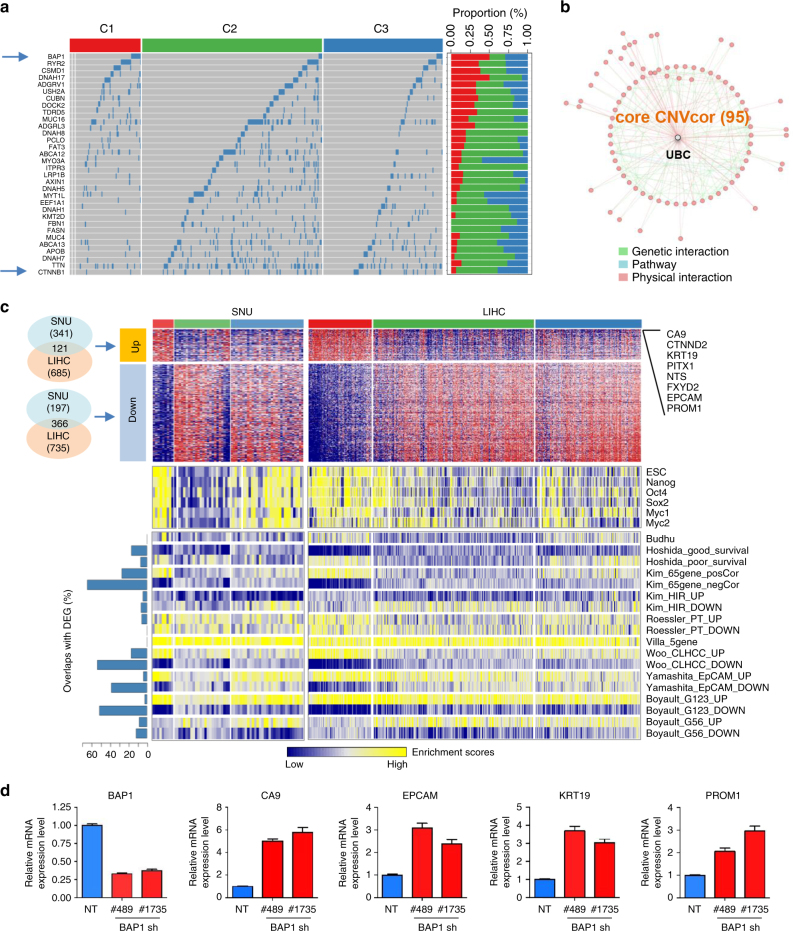
Identification of key molecular features for HCC subtypes. **a** Differentially mutated genes among the subtypes of LIHC are shown. A total of 37 differentially mutated genes are identified, which have differential mutation rates in C1 tumors compared to C2 or C3 tumors, respectively. The two samples of TCGA-G3-A25X-01 and TCGA-BC-4072-01 with no mutation data were excluded in the analysis (indicated with a right grey color). **b** Network of CNVcor genes is constructed using physical and genetic interactions from GeneMania software, which identifies the highest interconnecting hub gene, UBC. **c** Heatmaps show the differentially up- (DEG_UP, *n* = 121) and down-expressed (DEG_DOWN, *n* = 366) genes that are commonly found in both SNU and LIHC data sets, respectively (top). Top-ranked DEGs are indicated. The DEGs for SNU data are defined with fold difference >1.4 in comparison of iCl1 tumors with iCl2 or iCl3 tumors, while the DEG for LIHC data are defined with fold difference >2 and permuted Student’s *t* test *P* < 0.001 in comparison of the C1 tumors compared to C2 or C3 tumors, respectively. Gene set enrichment scores of the embryonic stem cell (ESC)-related gene sets (middle) and the nine molecular classifiers for HCC (bottom) are shown in SNU and LIHC data sets, respectively. Stemness gene sets of ESC, Nanog, Oct4, Sox2, Myc1, and Myc2, and the nine different HCC classifiers are obtained from previous studies as described in “Methods.” **d** Huh7 cells stably expressing non-targeting (NT) shRNA and *BAP1* shRNA (#489 or #1735) were established. Real time qPCR for *BAP1* and stemness genes (*CA9*, *KRT19*, *EPCAM*, and *PROM1*) was performed. The expression values represent means ± S.E.M. of three independent experiments

Next, we examined the mutation profiles of LIHC to determine whether they were associated with our subclassification. After filtering out the synonymous mutations, we obtained gene-level mutations of missense and nonsense variants. Overall, mutation frequencies did not differ among the subtypes (Supplementary Fig. 3). Among the 189 recurrently mutated genes with more than 10 mutations across the samples, we identified 37 differentially mutated genes, which had >5% difference of the mutational frequencies among the subgroups of C1, C2, and C3 (Fig. 5a). Interestingly, *BAP1* was the most frequently mutated gene in C1 (*n* = 10, 14.7%), but it was mutated less frequently in C2 (*n* = 4, 2.25%) and C3 (*n* = 6, 5.12%). *BAP1* expression is associated with multiple tumor types and high tumor phenotype penetrance^16^. By contrast, *CTNNB1* mutation was more frequent in the favorable prognostic groups (C2, *n* = 18, 10.16%; C3, *n* = 13, 11.11%), but less frequent in the aggressive subtype C1 (*n* = 2, 2.91%). Supporting this, previous studies have shown that the *CTNNB1* mutation is associated with favorable HCC prognosis^17^. Taken together, these findings suggest that molecular HCC subtypes associated with DNA copy numbers and DNA methylation are also linked to *BAP1* and *CTNNB1* mutations, which might play regulatory roles in subtype progression of HCC.

In addition, to address the functional determinants of the subtypes, we performed a network analysis using the core CNVcor genes (*n* = 95) and METcor genes (*n* = 179) shared between the SNU and LIHC data. Interestingly, the *UBC* gene was highly connected by physical and genetic interactions to most of the core CNVcor genes (77 out of 95, 81%), although *UBC* itself was not a member of CNVcor genes (Fig. 5b). *UBC* encodes ubiquitin C, which regulates multiple biological functions, and its expression is associated with tumor progression^18^. This suggests that dysregulation of CNVcor genes could be effectively suppressed by modulating ubiquitination, although further studies would be required to investigate this possibility in detail. The core METcor genes did not have a significant hub gene in this network analysis.

Furthermore, to delineate key distinctive features of the subtype transcriptomes, we identified differentially expressed genes (DEGs) among subtypes that are commonly found in both SNU and LIHC data sets, including up- (*n* = 121) and down-expressed (*n* = 366) genes. Notably, we identified that *CA9* was the top-ranked gene that was differentially expressed in iCl1/C1 tumors in comparison with other tumor subtypes (Fig. 5c, top). Indeed, *CA9* is a marker of hypoxia, and its overexpression is a poor prognostic marker in HCC^19^. In addition, in comparison with the other subtypes, the aggressive iCl1 and C1 tumors expressed high levels of stemness-related genes such as *KRT19*, *EPCAM*, and *PROM1*. Consistent with this, a recent study reported that *CA9* expression is associated with stemness-related phenotypes in HCC^20^. These results indicate that the aggressiveness of iCl1 and C1 tumors might be associated with expression of stemness traits. Based on this finding, we also evaluated the known stemness gene sets (i.e., gene sets for ESC, Nanog, Oct4, Sox2, Myc1, and Myc2). This analysis revealed that the iCl1 and C1 tumors exhibited significant differential expression of stemness genes in comparison with the other subgroups (Fig. 5c, middle). *CA9* expression might represent a promising candidate marker for the most aggressive subtype of tumors (iCl1 or C1).

We also compared our classification with the previous studies that have defined molecular classifiers for HCC subtypes. By applying nine different molecular classifiers as described in “Methods,” we evaluated their enriched expression in SNU and LIHC data sets, respectively. When we compared each of the HCC classifier with the DEGs (*n* = 487), only a subset of the HCC signature genes were overlapped with DEGs, suggesting that each classifier represents distinct biological characteristics (Fig. 5c, bottom left). However, Kim_65 genes (34 out of 65), Yamashita_EpCam_DOWN (7 out of 18), Woo_CLHCC_DOWN (203 out of 374), and Boyault_G123_DOWN (26 out of 50) genes showed substantial overlaps with the DEGs (>20%), indicating their expression similarity of the subtypes from those studies. Although not all the classifiers were matched to our classification, most of them exhibited differential enrichment among the subtypes. Particularly, C1 and iCl1 tumors exhibited differential expression of the prognostic signatures compared to the other subgroups, including Hoshida et al., Kim_65 genes, Roessler_PT_UP, Woo_CLHCC, Yamashita_EpCAM, and Boyault_G123 signatures (Fig. 5c, bottom right). This also imply that the expression of these previous classifiers might be associated with the frequencies of the aberrations of CNVcor and METcor genes. Collectively, we suggest that the C1 and iCl1 tumors might share functional features of those subtypes such as aggressiveness or stemness.

As shown in Fig. 5a, c, *BAP1* mutation is linked with the expression of stemness in C1 tumors. To clarify this association, we evaluated the effects of *BAP1* suppression on the expression of stemness genes in liver cancer cells. Knockdown of *BAP1* expression by transfecting the *BAP1*-targeting short hairpin RNAs (shRNAs) in Huh7 cells significantly upregulated the expression of stemness genes including *CA9*, *KRT19*, *EPCAM*, and *PROM1* (Fig. 5d). These results strongly support our finding that the *BAP1* mutation may contribute, at least in part, to the progression of an aggressive HCC subtype expressing stemness genes.

## Discussion

Previous studies demonstrated that integrative analysis of multi-layered genomic features of cancers could identify molecular subtypes, providing novel mechanistic and clinical insights into tumor heterogeneity, as well as revealed candidate therapeutic targets and biomarkers. However, unveiling the enormous complexity of cancer genome data remains challenging. Indeed, HCC has heterogeneous etiological backgrounds, which may also impede extraction of biologically meaningful information from genomic data. With this concern in mind, in this study, we enrolled patients who had a homogenous etiological background of hepatitis B virus (HBV) infection and had experienced the same clinical management in one hospital. As a strategy for integrating multi-layered genomic and epigenomic data, we defined the genomic and epigenomic deregulators of CNVcor and METcor genes, and demonstrated that these correlated genes could successfully identify subtypes of HCC that reflected their distinct, multi-layered molecular features, as well as their distinct prognostic outcomes. Our findings were validated in an independent TCGA data set, notwithstanding its heterogeneous sample composition. Moreover, we found that HCCs with higher frequencies of CNVcor aberration had higher frequencies of METcor aberration, demonstrating that that the patients who have frequent aberration of DNA copy numbers are prone to have frequent aberration of DNA methylation. This observation highlights the fact that aberrations of DNA copy number and methylation need to be considered together in data analysis.

Remarkably, our classification analysis based on CNVcor and METcor revealed novel molecular key features that represent new precision targets and biomarkers for HCC management. When we compared the mutation profiles among the subtypes, we observed differential mutation rates of *BAP1* and *CTNNB1*. In particular, C1 tumors had frequent mutations at *BAP1* (14.7%), although less frequent in overall HCC samples. *BAP1*, a tumor suppressor gene, encodes a nuclear deubiquitinase that is thought to mediate its effects through chromatin modulation, transcriptional regulation, and possibly the ubiquitin-proteasome system and DNA damage response pathway. Recently, mutations in *BAP1* have been detected in various malignancies, in which they confer increased susceptibility for tumorigenesis^21–24^. Recurrent *BAP1* mutations have also been observed in intrahepatic cholangiocarcinoma^25^, and a recent study showed that loss of *Bap1* in mice increases expression of H3K27me3 and Ezh2 while suppressing expression of *PRC2* targets^26^. Dysregulation of Polycomb genes is frequently observed in tumors expressing stemness traits^27^. This is consistent with our finding that C1 tumors had the top-ranked expression of stemness genes, including *CA9*, *KRT19*, *EPCAM*, and *PROM1* (encoding CD133) (Fig. 5c), well-known markers of stemness traits, as well as poorer prognostic outcomes in HCC^28–31^. Similarly, depletion of *BAP1* results in loss of differentiation and gain of stem-like properties in uveal melanoma cells^32^. In liver cancer cells, we also observed same results (Fig. 5d). These data raise a possibility that the aggressive behavior of C1 tumors might be mediated, at least in part, by *BAP1* mutation and the resultant induction of stemness traits.

In addition, by integrating our data with TCGA data, we identified *UBC* as a potential key regulator that acts as a hub gene for most CNVcor genes in the network. *UBC* encodes a component of the ubiquitin system whose activity yields free ubiquitin. The ubiquitin system has recently been identified as a target for cancer treatment^18, 33^, and *UBC* is implicated in diverse tumor types, contributing to both tumorigenesis and cancer progression^34, 35^. Thus, drugs targeting the ubiquitin/proteasome system, such as bortezomib, represent candidates for attenuating the aberrant regulation of CNVcor genes in aggressive iCl1/C1 tumors.

Besides the most aggressive subtype iCl1/C1, the favorable subtypes of iCl2/C2 and iCl3/C3 had no significant difference of prognostic outcomes. However, iCl2/C2 and iCl3/C3 subtypes showed distinct patterns on the levels of overall aberrations of DNA copy numbers and methylations (Fig. 2c and Fig. 3f, g). Further studies might be required to delineate underlying mechanisms for these discernible features of subtypes.

In conclusion, our systematic integration of genomic and epigenomic regulation of gene expression revealed coordinated multi-layered genomic aberrations of HCCs, and which could identify molecular subtypes of HCC providing novel mechanistic and clinical insights on the precision diagnostics and therapeutics for HCC patients.

## Methods

### Patients and tissue specimens

A total of 64 cases HCC patients with HBV infection from SNU hospital were enrolled in this study. The study was approved by the Institutional Review Board of SNU Hospital (IRB number, 1211-063-442) and obtained written informed consents from donors.

### Profiling of DNA copy numbers, DNA methylation, and mRNA expression

The frozen tissues from 64 cases of tumor specimens and 30 cases of the non-tumoral surrounding tissue specimens were used for genomic profiling. DNA copy-number variation (CNV), DNA methylation (MET), and mRNA expression (EXP) profiling in the same patients were performed using Human Omni1 chips, Infinium Human Methylation 27 BeadChip, and Human HT-12 Expression BeadChips as manufacturer’s instruction, respectively (Illumina, San Diego, CA, USA). Gene expression profile was normalized by log2 transformation, quantile normalization, and aggregated by HUGO official symbols. Each data profile was normalized by subtracting the average values per probe of the non-tumoral tissues to represent the fold differences between the tumor and non-tumoral tissues. Then, for CNV data, gene-level DNA copy numbers in each sample were mapped with the segmented CNV values by circular binary segmentation algorithm implemented in R package library “DNAcopy.” For DNA methylation profile, probe level *β*-values were filtered to remove the probes located on sex chromosomes. Then, the probes residing in CpG islands-related regions including CpG islands, Shelf, and Shore regions, differentially methylated regions, and gene promoter regions including the upstream 2500 bases from TSS, 5′UTR, and first-exon regions were mapped to their corresponding genes. For each of the processed profiles, the probes with >30 % of missing values across samples were removed, and the remained missing values were imputed by R package library “*impute*.”

For each data set, genomic coordinates of the probes in each data set were updated to human reference genome hg38 by using R package library “*liftOver*,” then the probes were matched to the corresponding probes of the EXP profiles. The tumor specific alteration was calculated by subtracting the average probe intensities of the non-tumoral tissues. After filtering out the probes with missing values >50% and the probes for sex chromosomes, data were imputed by s k-nearest neighbor algorithm. Then, pairwise Pearson’s correlation coefficients were calculated for each gene in the paired profiles of CNV vs. EXP and MET vs. EXP, respectively. In cases where multiple probes mapped to a gene, the probe with a mean or a minimum value of the correlation coefficients was used as a representative pair-matched probe for the CNV and MET profiles, respectively.

### Clustering analysis of multi-layered genomic profiles

NMF cluster analysis with standard “brunet” method and 50 iterations was employed to identify stable sample clusters using CNVcor and METcor genes, respectively. Number of clusters *k* was set to 2 to 5, and the preferred number of cluster was determined using the observed consensus map and cophenetic correlation between clusters, and the average silhouette width of the consensus membership matrix was determined by the R package “NMF.” The minimum number of a cluster member was set to 10. For integrated cluster analysis for CNV, MET, and EXP profiles, we applied “iCluster” method implemented in R package with default parameters and 50 iterations^15^.

### Validation of the molecular subtypes using TCGA data

Validation of the analysis results was performed using a data set of liver hepatocellular carcinoma (LIHC) from TCGA. Multi-layered profiles for DNA copy numbers, DNA methylation, mRNA expression, and mutations of LIHC data were obtained from official TCGA data portal (https://tcga-data.nci.nih.gov). After matching sample labels in each platform, we used 364 data sets that have the matched data sets of DNA copy numbers, methylation, and mRNA expression profiles. The data were preprocessed by applying same procedures used in our data set (SNU). For data integration of LIHC and SNU data, batch effects between the data sets were corrected using empirical Bayes methods implemented in an R Package “combat.” For mutation data analysis, we used 362 samples matched to the integrated data set excluding the two samples that did not have mutation profiles (i.e., TCGA-G3-A25X-01 and TCGA-BC-4072-01).

### Gene set enrichment and network analyses

For gene set analysis, the embryonic stem (ES) cell-related gene sets including ESC (ES cell), Nanog, Oct, Sox2, Myc1, and Myc2 were obtained from a previous study^36^. HCC classifiers from nine different studies were obtained at original publication sites, which included the gene signatures of Budhu et al.^37^, Villa et al.^38^, Hoshida_survival^39^, Yamashita_EpCam^40^, Kim_65 gene^41^, Kim_HIR (hepatic injury and regeneration)^42^, Boyault et al., Roessler_PT (portal vein thrombosis)^43^, and Woo_CLHCC (cholangiocarcinoma-like HCC)^30^ (Supplementary Table 1). For each sample, the enrichment of a gene signature was calculated by applying Kolmogorov–Smirnov test. Briefly, for each individual gene expression profile, two *P* values for the estimates D+ and D− were calculated by Kolmogorov–Smirnov test, which determines the significance of the directional (positive or negative) enrichment of distributions of the signature. The ES *S*_D+_ and *S*_D−_ for a given signature was calculated as −log10 (*P* value) from D+ and D−, respectively. The ES *S* was defined as *S*_D+_ if *S*_D+_ > *S*_D−_ and −*S*_D−_ if *S*_D+_ < *S*_D−_. In addition, canonical gene ontology analyses for biological processes were performed using DAVID software (https://david.ncifcrf.gov). Gene networks for given gene signatures were constructed using pathway, physical, and genetic interactions that were obtained from GeneMania plugin implemented in Cytoscape software (http://www.cytoscape.org).

### Cell culture and lentiviral vector transfection

The Huh7 HCC cell line was obtained from the Korea Cell Line Bank (KCLB; Seoul, Korea). 293TN cells were obtained from System Biosciences (Mountain View, CA, USA), and were cultured in DMEM supplemented with 10% FBS, 100 U/ml penicillin, and 100 µg/ml streptomycin. Lentiviral constructs expressing non-target (NT) and *BAP1* shRNAs were purchased from Sigma-Aldrich (SHCLNG-NM_004656; St. Louis, MO, USA), and transfected into 293TN cells (System Biosciences) with Lipofectamin 3000 transfection reagent (Invitrogen, Waltham, MA, USA). Two reaction tubes with 500 ul aliquots of opti-MEM (Invitrogen; A and B) were prepared. Tube A was added with 24 µl P3000 reagent and 8 ug of DNA (3 µg pGag-pol, 1 µg VSV-G, and 4 µg target plasmid). Tube B was added with 15 µl lipofectamin 3000. Then, the mixture of tube A to tube B was incubated for 5 min and added in small drops to the cells with 70–90% confluence. The particles were collected 2 days after transfection. The lentivirus-infected cells were puromycin-selected for 1 week and stabilized by culturing for 4 weeks.

### Real-time PCR

Cells were collected and total RNA was isolated using an RNeasy kit (Qiagen, Venlo, the Netherlands). The PrimeScript RT kit (Takara, Shiga, Japan) was used to reverse-transcribe the mRNA into complementary DNA. PCR was done using a CFX96 Real Time PCR Detection System (Bio-Rad Laboratories, Hercules, CA, USA) with Ssoadvanced Universal Supermixes (Bio-Rad). The sequences of primers were described in Supplementary Table 2. Analysis of each sample was performed at least three times for each experiment, and the relative expression levels were measured by average values of 2^−ΔΔCT^ ± S.D.

### Statistical analysis

All the statistical analysis was performed using the R package (http://www.r-project.org). OS was defined as the time from surgery to death. TTR was defined as the time from surgery date to the date of any diagnosed relapse. The follow-up time OS and TTR was truncated to 5 years.

### Data availability

The raw data of the genomic profiles are available in GEO database (http://www.ncbi.nlm.nih.gov/projects/geo) with an accession number GSE87630.

## Electronic supplementary material

Supplementary Data 1

Supplementary Information
