# Supplementary material for: Integrative analysis of genomic and epigenomic regulation of the transcriptome in liver cancer
Source: Nat Commun. 2017 Oct 10;8:839. doi: 10.1038/s41467-017-00991-w (PMC5635060; doi:10.1038/s41467-017-00991-w)
Supplement: Supplementary file 2 — Supplementary Information [file 41467_2017_991_MOESM2_ESM.pdf]

Description of Supplementary Files

File Name: Supplementary Information
Description: Supplementary tables and Supplementary Figures

File Name: Supplementary Data 1
Description: List of CNVcor and METcor genes

Supplementary Table 1. List of molecular classification signatures for HCC

Description	Gene signature name	Number of genes	Authors	Publication Year	PMID
5-gene signature	Villa	5	Villa et al	2015	25666192
17-gene metastasis predictor	Budhu	17	Budhu et al	2006	16904609
EpCAM-signature	Yamashita_EpCam_UP	53	Yamashita et al	2008	18316609
	Yamashita_EpCam_DOWN	18			
186-gene signature	Hoshida_good_survival	113	Hoshida et al	2008	18923165
	Hoshida_poor_survival	73			
65-gene signature	Kim_65gene_posCor	22	Kim et al	2012	22105560
	Kim_65gene_negCor	43			
233-gene signature for hepatic injury and regeneration (HIR)	Kim_HIR_UP	170	Kim et al	2014	25536056
	Kim_HIR_DOWN	63			
6 group classifier	Boyault_G123_UP	45	Boyault et al	2006	17187432
	Boyault_G123_DOWN	50			
	Boyault_G56_UP	12			
	Boyault_G56_DOWN	17			
Portal thrombosis signature	Roessler_PT_UP	108	Roessler et al	2010	21159642
	Roessler_PT_DOWN	53			
Cholangiocarcinoma-Like	Woo_CLHCC_UP	251	Woo et al	2010	20395200
	Woo_CLHCC_DOWN	374			

HIR; Hepatic injury and regeneration, PT; Portal vein thrombosis, CLHCC; cholangiocarcinoma-like Hepatocellular carcinoma

Supplementary Table 2. List of qRT-PCR primers for stemness genes

Gene		Primer sequences
BAP1	Forward	5`-CTACCACGACATCCGCTTCA-3`
	Reverse	5`-CAGGCAGCTGTGACTCTTGA-3`
CA9	Forward	5`-TACAGCTGAACTTCCGAGCG-3`
	Reverse	5`-CTAGGCTCCAGTCTCGGCTA-3`
KRT19	Forward	5`-AGATTGAACCGGGAGGTCG-3`
	Reverse	5`-CCTGATTCTGCCGCTCACTA-3`
EPCAM	Forward	5`-GTGCTGGTGTGTGAACACTG-3`
	Reverse	5`-GAAGTGCAGTCCGCAAACTT-3`
PROM1	Forward	5`-TCACCAGCAACGAGTCCTTC-3`
	Reverse	5`-GGTTTGCACGATGCCACTTT-3`

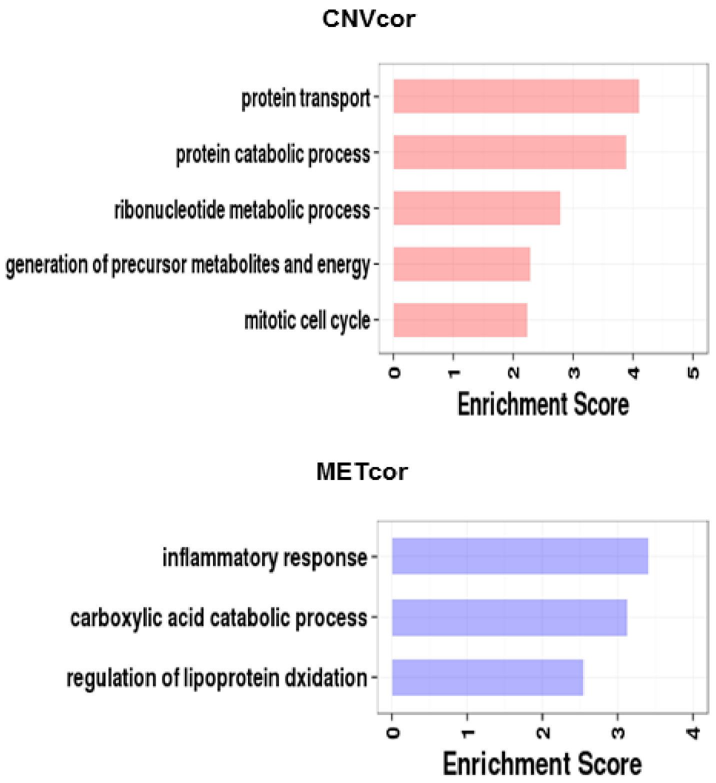

Supplementary Figure 1. Gene ontology analysis of CNVcor and METcor genes

Gene ontology (GO) analyses are performed using CNVcor (top) and METcor (bottom) genes using DAVID software (https://david.ncifcrf.gov). Enrichment scores of GO terms are shown.

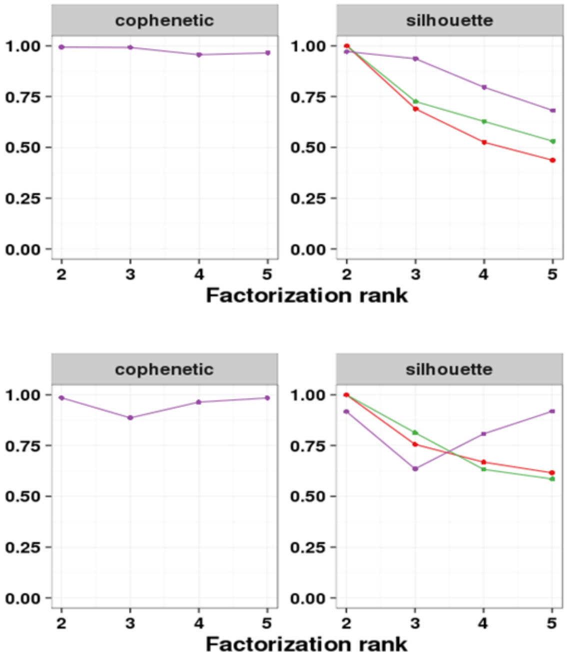

Supplementary Figure 2. NMF clustering analysis of CNVcor and METcor genes

NMF cluster analyses are performed for CNVcor (A) and METcor (B) genes. The cluster numbers of K=2,3,4,5 are examined and their cophenetic correlation between clusters and the average silhouette width of the consensus membership matrix as determined by the R package ‘NMF’.

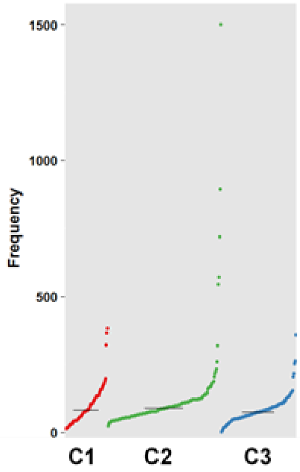

Supplementary Figure 3. Mutation frequencies among the HCC subgroups

Mutation frequencies of individual samples among the subtypes of C1, C2, and C3 are shown. Median of the mutation frequencies in each subtype are indicated by horizontal lines.
